# Blue LED-Associated Morphological Changes and Gene Expression Responses in *Trichoderma harzianum*

**DOI:** 10.3390/biology15161390

**Published:** 2026-08-14

**Authors:** Dong-Ryeol Yu, Min Seo Jung, Youn Jin Park, Myoung-Jun Jang

**Affiliations:** Department of Plant Resources, Kongju National University, Yesan 32439, Republic of Korea

**Keywords:** *Trichoderma harzianum*, blue light response, mycelial growth, conidiation, carbohydrate-active enzymes (CAZyme)

## Abstract

As LEDs are increasingly used in mushroom production, understanding the blue-light response characteristics of the pathogenic fungus *Trichoderma harzianum* is important for mushroom cultivation management. In this study, *T. harzianum* was cultured under blue LED conditions (460 nm), with darkness as the control, and its growth, morphology, and transcriptomic responses were compared. Blue LED exposure reduced colony diameter by 21.6% and 25.2% on days 1 and 2 of cultivation, respectively, and induced sporulation on day 3. RNA-seq Trinity gene-level features were quantified, and 23,086 and 34,839 genes showed higher and lower expression under blue LED illumination, respectively. Gene Ontology (GO) analysis of candidates selected using the criteria TPM ≥ 15 and |log2FC| ≥ 1 showed that the candidates with increased expression blue LED illumination were associated with transport and xenobiotic transport, whereas the candidates with decreased expression were associated with filamentous growth, cell wall polysaccharide biosynthetic process, and UDP-glucosyltransferase activity. Among the highly responsive candidates selected using the criteria TPM ≥ 15 and |log2FC| ≥ 3, carbohydrate-active enzyme (CA-Zyme)-related genes were identified, including glycosyltransferase family (GT) 8, GT35, glycoside hydrolase family (GH) 16, GH18, GH76, and multicopper oxidase. In contrast, the candidates with decreased expression included GT2, GT20, GT35, carbohydrate esterase family 5, and GMC oxidoreductase. RT-qPCR analysis of the selected candidate genes supported the RNA-seq-based expression trends. In addition, in the RNA-seq dataset, the expression levels of the blue light response-related genes BLR-1, BLR-2, and ENV1 increased by 2.8-, 1.9-, and 4.2-fold, respectively. These findings provide phenotypic and molecular candidates for future functional studies of the blue-light response in *T. harzianum*.

## Simple Summary

Blue light acts as an important environmental signal that can affect fungal growth and development, and various studies have investigated fungal responses to blue light. In particular, photoreceptors that perceive blue light have been relatively well studied; however, transcriptome-level expression patterns linking light perception to mycelial growth and morphological changes remain limited. In this study, *Trichoderma harzianum*, a fungus with biological and agricultural importance, was cultured under blue LED illumination and dark conditions to examine mycelial growth, spore formation, and transcriptome expression patterns using RNA sequencing. Under blue LED illumination conditions, mycelial growth was significantly reduced compared with darkness, whereas spore formation was promoted. Candidate genes showing expression changes under blue LED illumination conditions were identified, and selected candidates were further examined by RT-qPCR. Some candidate genes related to carbohydrate metabolism, cell-wall-associated processes, and light-response pathways showed altered expression patterns. These results suggest that LED exposure may be associated with both morphological changes and transcriptome responses in *T. harzianum*. This study provides candidate-gene information that may be useful for future studies on fungal light responses, developmental regulation, and environmental adaptation.

## 1. Introduction

Light is recognized as one of the most important external signals used by fungi to interact with their surrounding environment. Changes in light conditions induce diverse physiological and morphological responses in fungi, including alterations in growth rate, sporulation, morphological development, and secondary metabolite production [1,2]. Light perception in fungi is mediated by photoreceptors that respond to specific wave-lengths, and light responses have been extensively studied in many filamentous fungi [3,4]. In particular, blue light is known as an environmental signal that can induce mycelial growth inhibition, promotion of sporulation, metabolic changes, and stress-related responses in various fungi [5,6,7]. Blue-light responses are mainly regulated through the White Collar Complex photoreceptor system, and in *Trichoderma* spp., the BLR-1/BLR-2 complex, which is homologous to the White Collar Complex, plays a central role [8,9].

*Trichoderma* spp. have been widely used as biological control agents for the management of plant-pathogenic fungi, and their responses to various environmental factors have been actively investigated [10]. However, in mushroom cultivation, *Trichoderma* spp. have been identified as major causal agents of green mold disease, and several species, including *Trichoderma harzianum*, have been reported to cause disease in the production of *Agaricus bisporus* and *Pleurotus ostreatus* [11,12,13]. Notably, the mushroom-pathogenic lineages formerly referred to as *T. harzianum* biotypes Th2 and Th4 were subsequently reclassified as *T. aggressivum* f. *europaeum* and *T. aggressivum* f. *aggressivum*, respectively [14]. Recently, artificial light and LED treatments have been applied in edible mushroom cultivation to regulate mushroom productivity, quality, and physiological characteristics [15,16]. Because such light environments may affect not only the production of high-quality mushrooms but, also the growth and sporulation of pathogenic fungi such as *Trichoderma* spp., understanding the light responses of *Trichoderma* spp. in mushroom cultivation systems is important. Indeed, light has been reported to regulate physiological responses in *Trichoderma* spp., including conidiation, secondary metabolite production, and enzyme activity [17,18].

In *Trichoderma* spp., blue-light responses following light perception through the BLR-1/BLR-2 complex are known to be regulated not only by a single photoreceptor but, also through integration with multiple signaling pathways, including ENVOY. ENVOY has been proposed as a factor linking blue-light responses with nutrient signaling and cAMP regulation, and the HOG-MAPK pathway and its downstream transcription factor ATF1 have also been reported to participate in the regulation of blue-light responses, development, and oxidative stress responses [19,20,21,22]. These studies demonstrate the presence of an upstream signaling network in the blue-light response of *Trichoderma* spp.

Morphological changes in fungi are closely associated with changes in the expression of various enzymes directly involved in the synthesis and degradation of cell wall components [23,24]. Carbohydrate-active enzymes (CAZymes), including glycosyltransferases (GTs), glycoside hydrolases (GHs), carbohydrate esterases (CEs), and auxiliary activities (AAs), play key roles in cell wall formation and remodeling [25,26,27]. Recent studies have reported that light stimulation can alter the expression patterns of fungal CAZyme enzyme families, thereby regulating physiological and morphological characteristics [28,29,30]. However, it remains unclear which gene expression changes underlie blue-light-dependent mycelial growth and morphological changes in *T. harzianum*. Therefore, in this study, the effects of blue LED illumination on mycelial growth and sporulation-associated morphological changes in *T. harzianum* were analyzed using darkness as the control condition. To identify transcriptome-level expression changes that may be associated with blue LED-induced morphological changes, RNA-seq data from dark and blue-light conditions were used to perform Trinity-based de novo transcriptome assembly and fold-change-based expression analysis. Furthermore, candidate genes showing altered expression under blue LED illumination were selected, and the expression changes in major candidate genes were validated by RT-qPCR.

## 2. Materials and Methods

### 2.1. Fungal Strain and Identification

*Trichoderma harzianum* KCCM 60289 (ATCC 52445), used in this study, was obtained from the Korean Culture Center of Microorganisms (KCCM, Seoul, Republic of Korea). For molecular identification of the strain, genomic DNA was extracted using the DNeasy Plant Mini Kit (Qiagen, Hilden, Germany), and the RNA polymerase II large subunit 2 (rpb2) region was amplified using the fRPB2-5F and fRPB2-7cR primer pair (Table 1) [31]. PCR amplification was performed using the Bio-Rad MyCycler™ Thermal Cycler System (Bio-Rad, Hercules, CA, USA) and Taq DNA polymerase (Bioneer, Daejeon, Republic of Korea). The PCR conditions consisted of an initial denaturation at 98 °C for 15 min, followed by 38 cycles of denaturation 95 °C for 20 s, annealing at 50 °C for 40 s, and extension at 72 °C for 1 min 30 s, with a final extension step at 72 °C for 5 min. The amplified PCR products were subjected to Sanger sequencing by SolGent (Daejeon, Republic of Korea). The obtained rpb2 sequence was compared with sequences of *Trichoderma* species using NCBI BLASTn (2.17.0+) to confirm sequence similarity. For phylogenetic analysis, the rpb2 sequence obtained in this study and reference sequences retrieved from GenBank (https://www.ncbi.nlm.nih.gov/genbank/, accessed on 9 June 2026)were used together, and multiple sequence alignment was performed using Multiple Alignment using Fast Fourier Transform (MAFFT) v.7 [32]. A phylogenetic tree was then constructed in MEGA version 12.1 using the Neighbor-Joining method [33]. Evolutionary distances were calculated using the Taji-ma-Nei model [34], the reliability of the tree was evaluated by bootstrap analysis with 500 replicates [35], and gaps and missing data were treated using the pairwise deletion option.

### 2.2. Culture Condition and Blue LED Treatment

The strain was cultured on potato dextrose agar (PDA; BD Difco, Sparks, MD, USA) at 25 °C. To reactivate fungal growth, the culture was subcultured twice, and the second subculture showing the highest growth vigor was used for subsequent experiments. The cultures were incubated for a total of 5 days, and light exposure was maintained throughout the incubation period. All treatments were performed with five replicates, and during incubation, photographs were taken at the same time each day and colony diameter was measured using vernier calipers (Mitutoyo, Kawasaki, Japan). Darkness was used as the control condition, and blue LED illumination was provided using a custom-assembled LED panel with a wavelength range of 450–495 nm and a peak wavelength of 460 nm. Blue light treatment performed in a separate enclosed box placed inside a dark incubation room. The boxes were shielded with blackout curtains, and the inner surfaces were matte black to minimize light reflection and cross-illumination. Light intensity was measured at the culture (plate) surface as photosynthetic photon flux density (PPFD, µmol m^−2^ s^−1^) and the PPFD of blue light was 53 µmol m^−2^ s^−1^. The distance between the LED source and the agar surface was fixed at 20 cm for all treatments. Petri dishes were incubated with lids in place throughout the incubation period. Relative humidity was not actively controlled and remained at the ambient level of the incubation room/boxes.

### 2.3. RNA Extraction and Sequencing

To compare morphological responses over a 5-day cultivation period and transcript abundance profiles following continuous blue LED exposure, mycelia were harvested at the predefined endpoint, day 5, from five independently cultured plates for each condition. Mycelia collected from the five plates within each condition were pooled to generate one composite sample representing the corresponding culture condition. Total RNA was extracted from each composite sample using the HiGene™ Total RNA Prep Kit (BioFACT, Daejeon, Republic of Korea) according to the manufacturer’s protocol. Consequently, one pooled RNA sample and one RNA-seq library were generated for each condition (*n* = 1 pooled library per condition). The extracted total RNA was submitted to e-biogen (Seoul, Republic of Korea) for sequencing. RNA quality was assessed using the TapeStation 4000 system, and cDNA libraries were constructed using poly(A) RNA selection and the CORALL RNA-seq V2 Library Prep Kit (Lexogen GmbH, Vienna, Austria). Sequencing was then performed on the Illumina NextSeq 2000 platform in a paired-end 150 bp format, generating more than 6 Gb of data per sample.

### 2.4. Trinity-Based De Novo Transcriptome Assembly, Expression Estimation, and Reference-Based Annotation

The obtained raw paired-end FASTQ reads were preprocessed using fastp v1.3.3. Read preprocessing was performed using a minimum quality score of 20 and a minimum read length of 36 bp, and low-quality bases and short reads were removed. Given that the available reference genome of *T. harzianum* CBS 226.95 does not correspond to the exact strain used in this study, a de novo transcriptome assembly strategy was adopted to re-duce potential bias caused by strain-level sequence divergence and to improve transcript recovery from the RNA-seq reads. The clean paired-end reads from the two condition-specific libraries were combined solely for de novo transcriptome assembly using Trinity v2.15.2. The assembled Trinity transcriptome was subsequently used as the reference for transcript abundance estimation.

After generating a Salmon index for the Trinity-assembled transcriptome, transcript abundance was independently quantified for the dark and blue LED libraries using the corresponding clean reads. Transcript expression levels were calculated as transcripts per million (TPM), and transcript-level abundance estimates were summarized into a Trinity gene-level abundance matrix. The mapping rate of each library was examined as a quali-ty control metric between the reference and the sequencing reads.

To assess the coding potential of the assembled transcriptome, open reading frames (ORFs), coding sequences, and predicted protein sequences were predicted using TransDecoder v5.7.1. The predicted sequences were compared against the *T. harzianum* CBS 226.95 reference protein database using DIAMOND blastp, and reference-based annotation of Trinity transcripts was performed based on the best-hit information. The sequencing data generated in this study were deposited in the NCBI Gene Expression Omnibus (https://www.ncbi.nlm.nih.gov/geo/, accessed on 4 June 2026) under accession number GSE334040.

### 2.5. Comparative Transcript Abundance Profiling, Fold-Change-Based Candidate Screening, Functional Annotation, and GO Term Classification

To compare the overall expression patterns, the Trinity gene-level abundance matrix generated from Salmon quantification was used, and expression directionality was examined by comparing TPM values between the dark and blue LED illumination without applying an additional cutoff. To characterize the functional properties of the candidate gene sets, Gene Ontology (GO) annotations were assigned to the protein sequences predicted by TransDecoder using eggNOG-mapper. GO enrichment analysis was then performed for candidate genes selected using the criteria TPM ≥ 15 and |log2FC| ≥ 1 with custom Python scripts (v3.12.11). The TPM ≥ 15 threshold was used as an abundance filter to reduce the influence of potentially unstable fold-change estimates from low-abundance transcripts, and genes passing the TPM ≥ 15 and |log2FC| ≥ 1 filters were interpreted as candidates showing expression changes under blue LED illumination. Enrichment analysis was conducted using Fisher’s exact test, and *p*-values were adjusted for multiple testing using the Benjamini–Hochberg method to calculate FDR values. Representative GO terms were selected based on the criteria FDR < 0.05, test_count ≥ 5, enrichment ratio > 1, and identifiable GO names, while unmatched GO terms and obsolete terms were excluded. To select genes showing strong expression changes under blue LED illumination, an additional criterion of TPM ≥ 15 and |log2FC| ≥ 3 was applied. This highly responsive candidate set was used to identify representative genes potentially associated with morphological changes and to select targets for RT-qPCR validation.

In addition, to identify transcripts related to photoreceptor-associated candidates, photo-receptor-related keywords were searched in the eggNOG annotation results, and the TPM and log2FC values of each candidate were confirmed using the Trinity gene-level abundance matrix.

### 2.6. Reverse Transcription-Quantitative PCR

Reverse transcription-quantitative PCR (RT-qPCR) was performed to validate the ex-pression patterns of selected candidate genes identified through transcriptome analysis. cDNA was synthesized from RNA extracted from mycelia grown under each light condition using RT Pre-Mix (BIO-FACT, Daejeon, Republic of Korea). RT-qPCR was conducted using a Bio-Rad CFX Connect Real-Time System (Bio-Rad, Hercules, CA, USA) with BioFACT™ 2X Real-Time PCR Master Mix (BIOFACT, Daejeon, Republic of Korea). The thermal cycling program was as follows: an initial denaturation at 95 °C for 15 min, followed by 42 cycles of denaturation at 95 °C for 20 s and annealing/extension at 60 °C for 40 s. A melt curve analysis was then performed from 65 to 95 °C with 0.5 °C increments (5 s per step). All primer pairs were designed in this study using Primer3 based on the target gene sequences. Gene expression levels were calculated using the 2^−ΔΔCt^ method with Actin1 as an internal control. The primers used are listed in Table 2.

### 2.7. Statistical Analysis

Colony diameter data are presented as the mean ± standard deviation, and statistical analyses were performed using R version 4.5.1 in RStudio, an integrated development environment for R. To analyze the interaction between light condition and incubation time, repeated-measures two-way analysis of variance (ANOVA) was conducted. Differences between the dark and blue LED illumination at each incubation day were tested using post hoc pairwise comparisons based on estimated marginal means (emmeans). Statistical significance was set at *p* < 0.05. Graphs of the statistical data were generated using the ggplot2 package.

## 3. Results

### 3.1. Phylogenetic Confirmation of Trichoderma harzianum KCCM 60289

**Figure 1:**
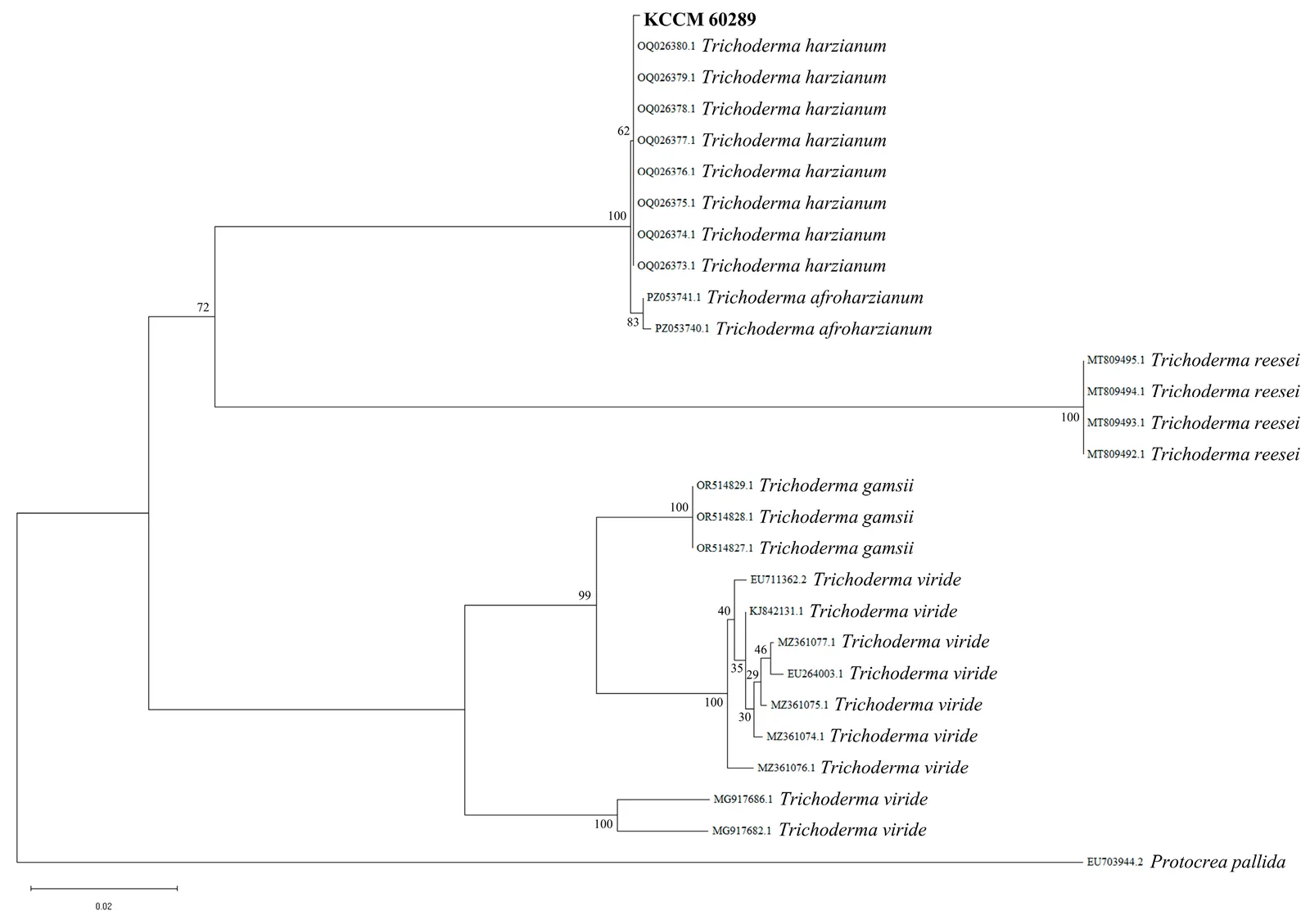
Phylogeny of the *Trichoderma* complex based on rpb2. The tree was constructed using reference sequence of *Trichoderma* species, with *Protocrea pallida* as the outgroup. Numbers at nodes indicate bootstrap values.

To confirm the phylogenetic position of the strain used in this study, phylogenetic analysis was performed based on the rpb2 sequence (Figure 1). As a result, KCCM 60289 (ATCC 52445) was positioned within the same clade as the *T. harzianum* reference sequences. In addition, it formed a phylogenetic group distinct from *T. reesei*, *T. gamsii*, *T. viride*, and *Protocrea pallida*, which was used as the outgroup.

### 3.2. Effect of Blue LED Illumination on Mycelial Growth

**Figure 2:**
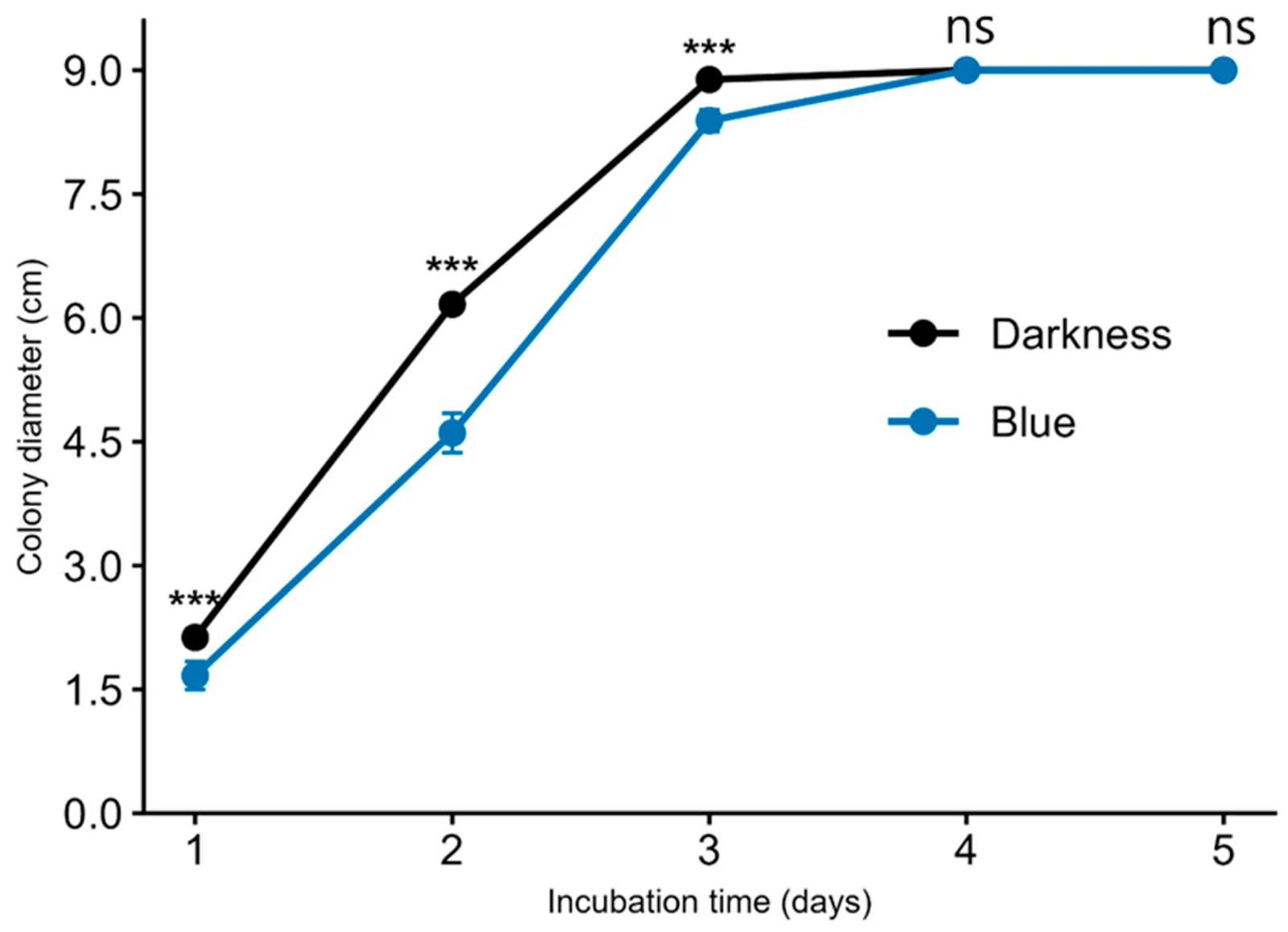
Colony diameter of *T. harzianum* under dark and blue LED illumination over five days. Data represent mean ± SD from five biological replicates. Asterisks indicate significant differences between dark and LED illumination at each time point based on post hoc comparisons following repeated-measures two-way ANOVA. *** *p* < 0.001.

Figure 2 presents the changes in colony diameter of *T. harzianum* cultured under dark and blue LED illumination for 5 days. After 1 day of cultivation, the colony diameter was 2.13 cm under the dark condition and 1.67 cm under the blue LED illumination, indicating slower mycelial growth under blue light. By day 2, the colonies had expanded to 6.16 cm under the dark condition and 4.61 cm under blue LED illumination, while mycelial growth remained suppressed under blue LED illumination. At day 3, the colony diameter reached 8.89 cm under the dark condition and 8.32 cm under blue LED illumination. From day 4 onward, colonies under both conditions fully reached the diameter of the 9 cm plate and maintained the same level until day 5, the endpoint of the experiment. Therefore, differences in growth between light conditions could not be distinguished at days 4–5 because the colony diameter had reached the plate diameter.

### 3.3. Promotion of Conidiation Under Blue LED Illumination

**Figure 3:**
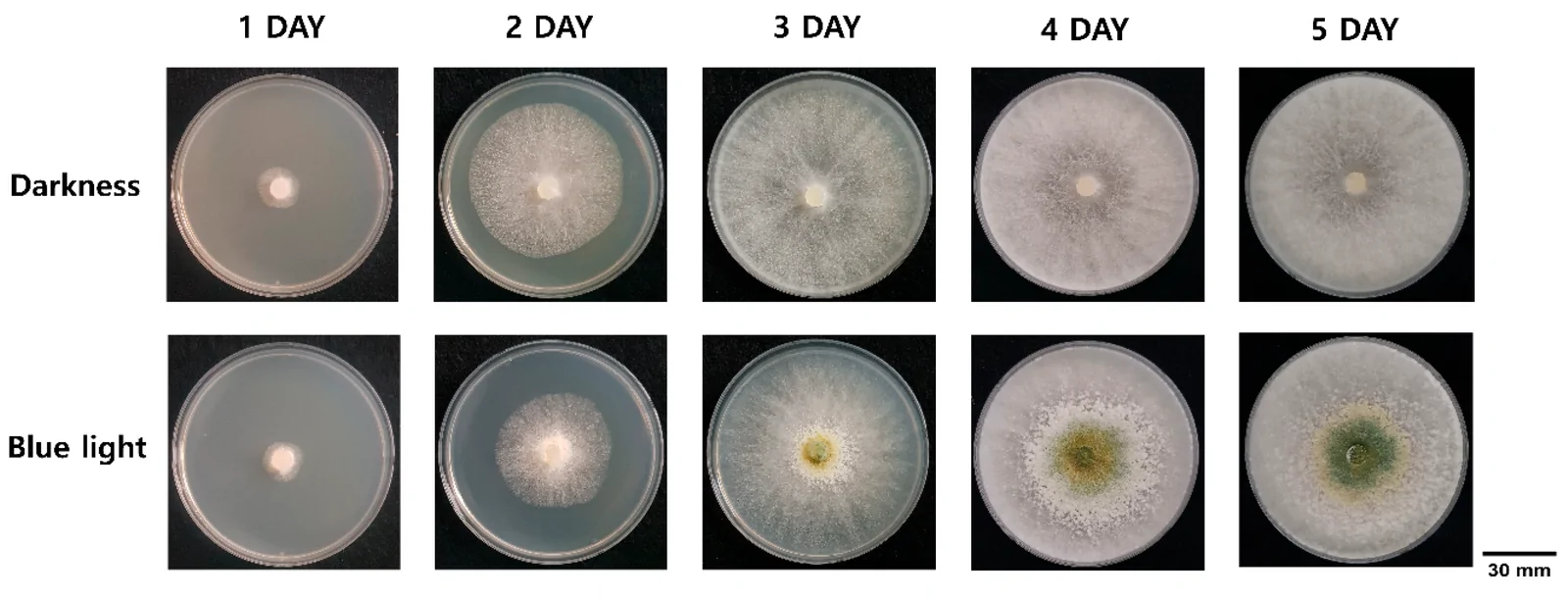
Time-course changes in mycelial growth and conidiation of *Trichoderma harzianum* cultured on potato dextrose agar (PDA) under dark and blue LED conditions for 5 days. Representative images were obtained daily from day 1 to day 5. Scale bar = 30 mm (applies to all panels).

The morphological characteristics of *T. harzianum* cultured under dark and blue LED illumination are presented in Figure 3. On days 1 and 2 of cultivation, no distinct morphological differences were observed between the dark and blue LED illumination, except for differences in mycelial growth. However, conidiation began from day 3 under the blue LED illumination and was continuously observed until the endpoint of the experiment. In contrast, under the dark condition, white mycelia continued to grow radially, and no distinct conidiation was observed until day 5.

### 3.4. Overall Transcriptome Expression Patterns Between Dark and Blue LED Illumination

**Figure 4:**
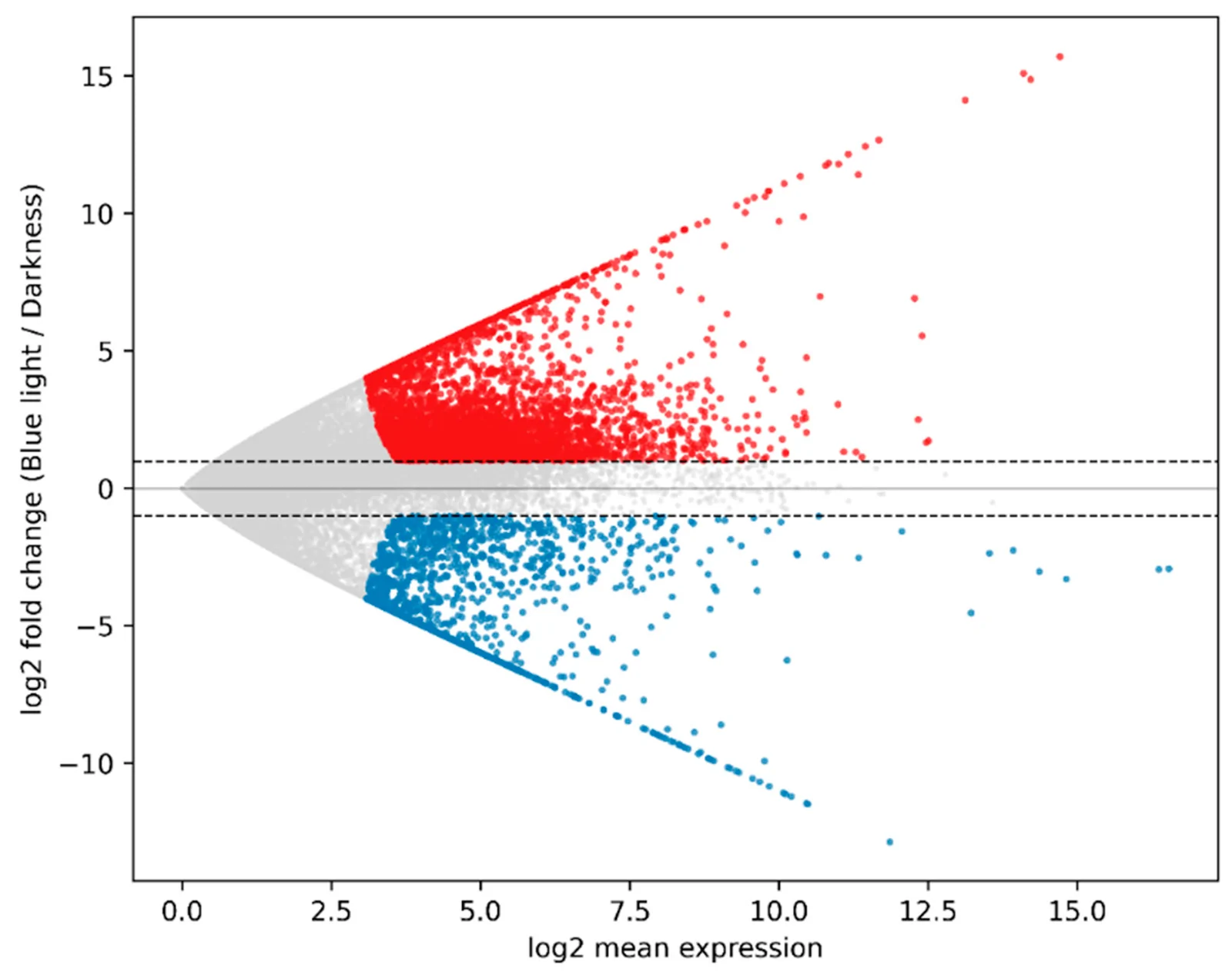
Transcriptome-wide expression patterns between dark and blue LED illumination. MA plot showing gene-level expression patterns of the Trinity transcriptome between dark and LED illumination. Each point represents a Trinity gene. The x-axis indicates log2 mean expression, and the y-axis indicates log2 fold change calculated as blue LED illumination /darkness. Red and blue points indicate genes located above the log2FC +1 reference line and below the log2FC −1 reference line, respectively, whereas gray points indicate genes within the log2FC ± 1 range. The dashed lines represent log2FC ± 1 reference lines for visualizing the magnitude of expression differences.

Quantification of RNA-seq reads from the dark and blue LED illumination against the de novo transcriptome resulted in Salmon mapping rates of 85.12% and 80.81%, respectively. The gene-level TPM matrix contained a total of 58,036 Trinity genes, and the overall expression distribution is presented in Figure 4. Under blue LED illumination, 23,086 genes showed higher expression, 34,839 genes showed lower expression, and 111 genes showed no change in expression level.

### 3.5. Gene Ontology (GO) Enrichment Analysis of Fold-Change-Based Candidate Gene Sets

Because broad expression differences between the dark and blue LED illumination were observed in the overall transcriptome expression profile, candidate genes responsive to blue LED illumination were selected from the 58,036 Trinity genes using the criteria |log2FC| ≥ 1 and TPM ≥ 15 for functional analysis. A total of 6035 candidate genes were identified, of which 4395 showed higher TPM values and 1640 showed lower TPM values under blue LED illumination. GO enrichment analysis of these candidates revealed that the higher-abundance candidate set was mainly enriched in GO terms related to localization, transport, xenobiotic response, and transmembrane transporter activity. In contrast, the lower-abundance candidate set was enriched in GO terms associated with filamentous growth, cell wall polysaccharide/β-glucan biosynthesis, fatty acid metabolism, glycosyltransferase activity, and glucan synthase activity (Table 3).

### 3.6. Selection of Candidate Genes Showing Marked Expression Changes Under Blue LED Illumination and Confirmation by RT-qPCR

**Figure 5:**
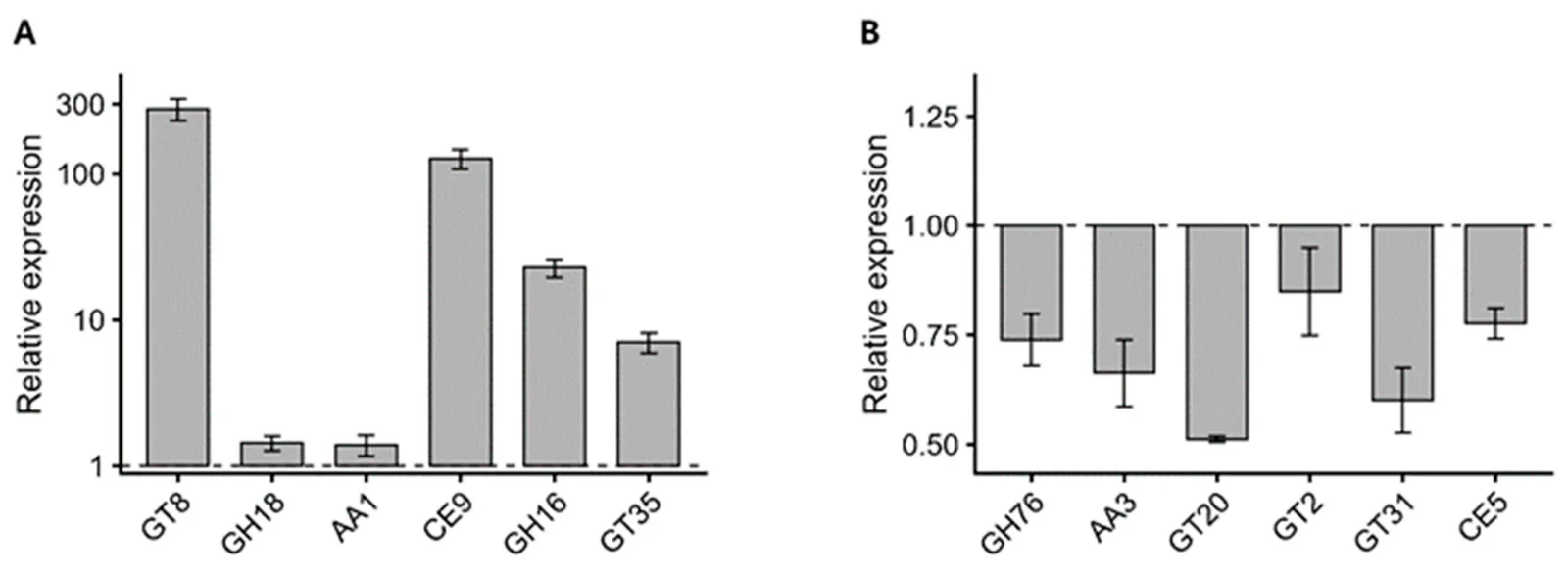
RT-qPCR analysis of selected candidates with pronounced expression changes under blue LED illumination. Relative expression levels of selected candidate genes under blue LED illumination compared with darkness. Candidate genes were selected from the fold-change-based transcriptome analysis using a |log2FC| ≥ 3 threshold. (**A**) Candidates classified as upregulated based on the fold-change-based RNA-seq screening. (**B**) Candidates classified as downregulated based on the fold-change-based RNA-seq screening. Relative expression was calculated using the 2^−ΔΔCt^ method, with the dark condition set to 1.0. bars indicate mean ± SD.

Based on the fold-change-based transcriptome analysis, candidate genes showing marked expression changes under blue LED illumination relative to the dark condition were selected using the criterion |log2FC| ≥ 3 (Table 4). The selected genes belonged to glycosyltransferase (GT), glycoside hydrolase (GH), carbohydrate esterase (CE), and auxiliary activity (AA) families and were mainly associated with CAZyme-related carbohydrate metabolism and cell wall-related functions. The expression patterns of these selected genes were further examined by RT-qPCR (Figure 5).

The upregulated candidates included GT8, GH18, AA1 multicopper oxidase, CE9, GH16, and GT35. Their log2FC values ranged from 3.182 to 4.584, with GT8 showing the highest increase in expression.

The downregulated candidates included GH76, AA3 GMC oxidoreductase, GT20, GT2, GT31, and CE5. Their log2FC values ranged from −3.44 to −7.32, with the AA3 GMC oxidoreductase candidate showing the greatest decrease.

RT-qPCR analysis showed that, among the upregulated candidates, GT8 exhibited the highest relative expression level, with a 275.64-fold increase, while CE9 also showed a strong increase of 126.53-fold. GH16 and GT35 showed increases of 22.63-fold and 7-fold, respectively, whereas GH18 and AA1 showed only weak increases of 1.40-fold and 1.39-fold, respectively. Among the downregulated candidates, all selected genes showed reduced relative expression levels. In particular, GT20 showed the greatest decrease, with a relative expression level of 0.51-fold. The other downregulated candidates, GH76, AA3, GT2, GT31, and CE5, showed relative expression levels ranging from 0.60- to 0.84-fold. Although the magnitude of relative expression changes confirmed by RT-qPCR differed from the fold-change values obtained from transcriptome analysis, the selected candidate genes maintained the predicted direction of upregulation or downregulation.

### 3.7. Expression Patterns of Blue-Light Receptor-Related Genes and Confirmation by RT-qPCR

To examine the expression patterns of genes associated with blue-light perception and response regulation under blue LED illumination, BLR-1, BLR-2, and ENV1 were analyzed. Transcriptome analysis showed increased expression of BLR-1, BLR-2, and ENV1, with log2FC values of 1.48, 0.92, and 2.07, respectively (Table 5).

**Figure 6:**
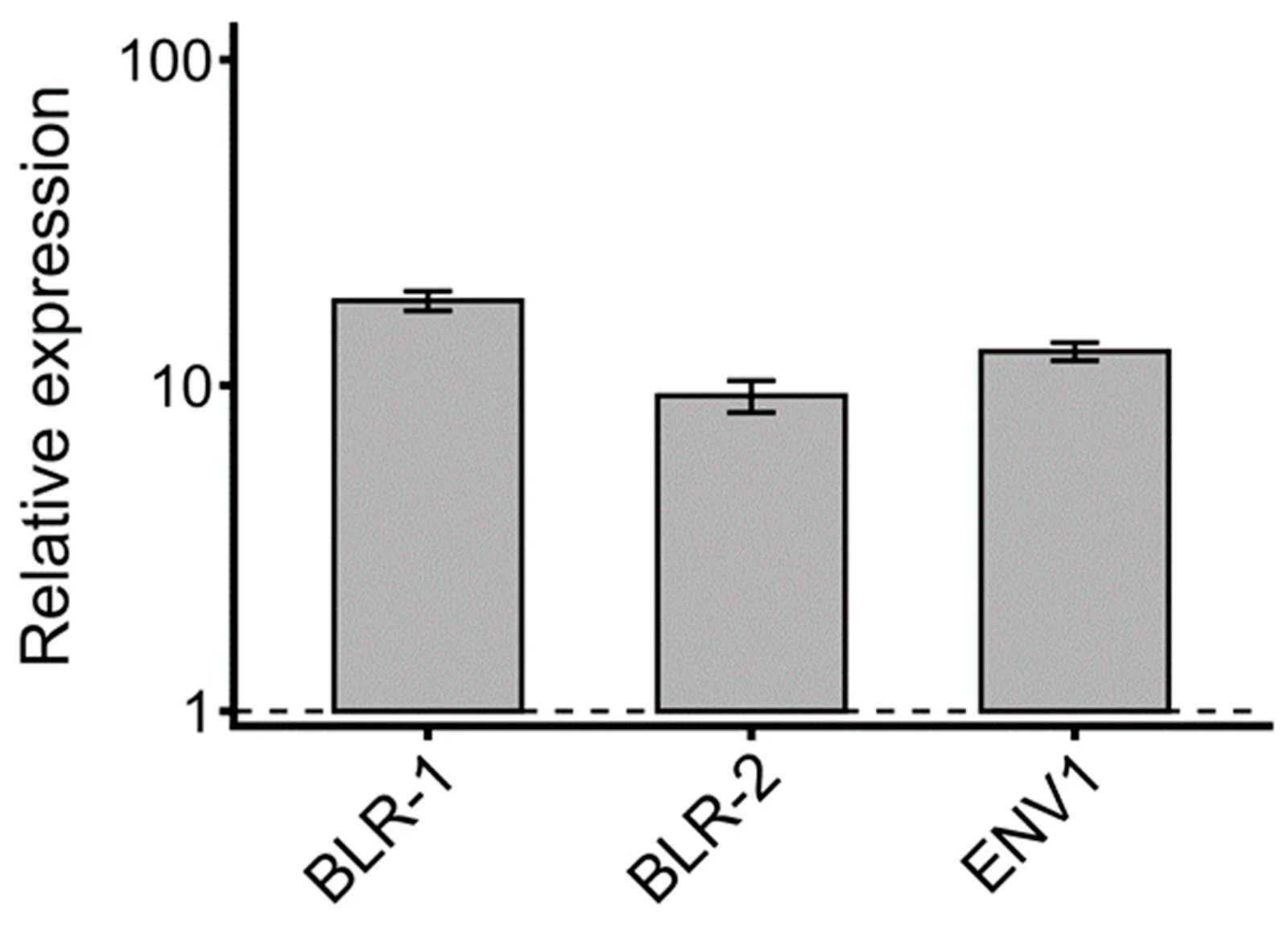
RT-qPCR analysis of photoreceptor-related genes under blue LED illumination. Relative expression levels of BLR-1, BLR-2 and ENV1 were analyzed under blue LED illumination compared with darkness. Relative expression calculated using the 2^−ΔΔCt^ method, with the dark condition set to 1.0. bars indicate mean ± SD.

RT-qPCR analysis also confirmed that all three genes exhibited higher relative expression under blue LED illumination than under the dark condition. BLR-1 showed the greatest increase, with a 18.2-fold elevation, while BLR-2 and ENV1 increased by 9.2-fold and 12.7-fold, respectively (Figure 6). Thus, BLR-1, BLR-2, and ENV1 showed consistent up-regulation under blue LED illumination.

## 4. Discussion

### 4.1. Blue LED Illumination Alters Hyphal Growth and Conidiation in T. harzianum

Light is an important environmental signal that regulates fungal growth, development, reproduction, and metabolism [36]. In particular, blue light is closely associated with asexual reproduction, morphological changes, and light-dependent transcriptional responses in fungal photobiology [1,36]. In many filamentous fungi, blue-light perception is linked to conidiation and developmental regulation, and recent research in *Trichoderma guizhouense* has further shown that blue light regulates conidiation and gene expression through BLR1-dependent transcriptional and alternative-splicing mechanisms [8,19,37].

Blue LED illumination inhibited early mycelial growth but promoted conidiation in *T. harzianum*, with sporulation becoming evident from day 3. This pattern suggests a shift in the balance between vegetative growth and asexual development. Similar effects of blue light on mycelial growth and conidiation have been reported in *Trichoderma* spp. [38,39]. In fungi, light may function not merely as a growth-inhibitory factor, but also as a developmental cue indicating that hyphae have reached the surface or an environment favorable for spore dispersal. Accordingly, the inhibition of early mycelial growth and promotion of conidiation observed under blue LED illumination in this study may reflect a developmental transition from vegetative growth to asexual reproduction. These observations may provide a basis for investigating light responses and green mold management in *T. aggressivum*, an important mushroom pathogen [14]. Because sporulation of *T. aggressivum* f. europaeum has been associated with reduced *Agaricus bisporus* mycelial growth and green mold development [40], the promotion of conidiation by blue LED illumination in *T. harzianum* suggests that light spectrum and exposure timing should be carefully evaluated rather than blue light being regarded as a direct control measure. Assessing the growth, conidiation, and pathogenicity of *T. aggressivum* under different light conditions may support light-management strategies complementary to heat treatment and disinfection [41].

### 4.2. Blue LED-Associated Transcriptome Expression Patterns and GO-Based Functional Shift

Although more genes showed lower abundance at the whole-transcriptome level, the filtered candidate set contained more higher-abundance than lower-abundance candidates under blue LED illumination. This difference suggests that blue light does not uniformly suppress the entire transcriptome but, instead exerts differential effects on gene sets that respond more distinctly to blue LED. Such bidirectional transcriptomic regulation is consistent with previous studies on light responses in *Trichoderma* [42]. Recent transcriptome studies have likewise reported broad light-dependent functional changes involving stimulus responses, DNA metabolism, cell-cycle regulation, metabolism, and other regulatory processes, supporting the GO-based shifts observed in the present study [43,44]. The enrichment of transport- and stimulus-response-related functions in the higher-abundance candidate set suggests transcriptional adjustment associated with membrane transport and responses to external stimuli. Similarly, recent transcriptome studies of fungal light responses and blue-light receptor function have identified broad functional changes involving stimulus responses, metabolism, DNA-associated processes, cell-cycle regulation, and other regulatory pathways [45]. These findings are broadly consistent with the GO-based functional shifts observed in the present study.

In contrast, the functional categories represented in the lower-abundance candidate set were enriched in GO terms associated with filamentous growth, cell wall polysaccharide/β-glucan biosynthesis, fatty acid metabolism, glycosyltransferase activity, and glucan synthase activity. The reduction in these functional categories may be linked to the inhibition of early mycelial growth. The fungal cell wall is a dynamic structure composed of glucans, chitin, glycoproteins, and other components, and it is essential for maintaining cell morphology, mechanical stability, environmental stress responses, and hyphal growth [46,47]. In particular, β-glucan biosynthesis plays an important role in cell wall formation and hyphal growth [48,49], while glycosyltransferases and glucanosyltransferase family enzymes may participate in the integration of cell wall glycoproteins and the chitin matrix [50,51].

### 4.3. CAZyme Candidate Genes with Marked Responses to Blue LED Illumination

The 12 candidate genes showing strong responses to blue LED illumination selected using the criterion |log2FC| ≥ 3 consisted of carbohydrate-active enzyme (CAZyme)-related genes belonging to the GT, GH, AA, and CE families. This is notable because it indicates that candidate transcripts showing strong responsiveness to blue LED illumination were concentrated in enzyme families associated with carbohydrate metabolism and cell wall-related functions. CAZymes comprise enzyme families involved in the formation, degradation, or modification of glycosidic bonds and are closely associated with the metabolism of carbohydrates and poly-saccharides [52,53]. These findings suggest that blue LED exposure was associated with carbohydrate metabolism and cell wall-related functions in *T. harzianum*. Blue-light-associated transcriptome studies in the basidiomycetes *Pleurotus eryngii*, *Lentinula edodes*, and *Pleurotus ostreatus* have also reported changes in the expression of genes related to CAZymes or carbohydrate metabolism following blue-light treatment [29,54,55].

Among the higher-abundance candidates supported by RT-qPCR, GH16 was of particular interest because it has been reported in *T. harzianum* as an endo-β-1,3-glucanase associated with cell wall biosynthesis and remodeling [56]. Thus, the increased expression of GH16 in this study may reflect transcript-level changes associated with glucan remodeling under blue LED illumination.

CE9 is associated with Glc-NAc-derived amino sugar metabolism, and in *T. reesei*, the GlcNAc catabolic gene cluster has been reported to be regulated by Regulator of N-acetylglucosamine catabolism (Ron1) [57]. Considering that Ron1-mediated chitin metabolism is associated with asexual development and cell-wall chitin integrity in *Beauveria bassiana* [58], the upregulation of CE9 observed in this study may be associated with chitin- and cell-wall-related processes accompanying morphological development under blue LED illumination.

In addition, GT35 also showed an increasing trend in RT-qPCR. The GT35 family is associated with α-glucan/glycogen phosphorylase, and fungal glycogen has been reported to function as a storage carbohydrate that is dynamically regulated during mycelial growth and development [27,59]. Therefore, the increased expression of GT35 suggests the possible reorganization of glycogen metabolism accompanying the morphological changes observed under blue LED illumination. In contrast, AA1 and GH18, which were selected as upregulated candidates in the fold-change-based transcriptome analysis, showed only weak increasing trends in RT-qPCR.

Conversely, among the downregulated candidates, GT20, GT31, and AA3 were notable. The GT20 family is associated with trehalose-6-phosphate synthase, and trehalose metabolism is known to influence not only storage carbohydrate metabolism but also oxidative and desiccation stress resistance and developmental processes in fungi. In particular, trehalose-6-phosphate synthase has been reported to be associated with oxidative and desiccation stress resistance in *Fusarium verticillioides* [60]. Therefore, the downregulation of GT20 may reflect transcript-level changes related to trehalose-associated storage carbohydrate metabolism and stress-protective metabolism under blue-light conditions. GT31 belongs to the β-1,3-glycosyltransferase family and is known to function in glycan and glycoprotein modification. In fungi, glycosylation is closely linked to growth, cell wall synthesis, and developmental processes [61]. The downregulation of GT31 may indicate a relative decrease in transcripts associated with specific glycosylation and glycan modification under blue-light conditions. AA3 GMC oxidoreductase functions in alcohol or carbohydrate oxidation and generates hydrogen peroxide or hydroquinone during the reaction process, thereby supporting the activity of other oxidases or glycoside hydrolases [62]. From this perspective, the downregulation of AA3 suggests that oxidative carbohydrate degradation or extracellular redox metabolism may have been relatively reduced under blue LED illumination.

Collectively, the nonuniform expression patterns of CAZyme-related candidates suggest that blue LED illumination was accompanied by selective transcript-level changes in genes associated with cell wall remodeling, amino sugar metabolism, storage-carbohydrate metabolism, glycan modification, and extracellular redox processes.

### 4.4. Expression of Photoreceptor-Related Genes Under Blue LED Illumination

Under blue LED illumination, BLR-1, BLR-2, and ENV1 showed clear increases in ex-pression in both transcriptome analysis and RT-qPCR. BLR-1 and BLR-2 are known as blue-light receptors homologous to WC-1/WC-2 in *Neurospora crassa* and have been re-ported as key components required for the regulation of photoconidiation and mycelial growth in *T. atroviride* [8]. In particular, overexpression of BLR-2 has been reported to in-crease blue-light sensitivity and photoconidiation [63]. More recently, a study in *T. gui-zhouense* demonstrated that BLR-1 and BLR-2 are essential for blue-light-induced sporulation and that the BLR-1/BLR-2 complex acts as a central regulator of blue-light-responsive gene regulation together with the HOG pathway [21]. The increased expression of BLR-1 and BLR-2 observed in this study is consistent with the expression response of a conserved blue-light signaling module reported in other *Trichoderma* species and may be associated with the inhibition of mycelial growth and the promotion of sporulation under blue LED illumination.

The increased expression of ENV1 is also noteworthy. ENV1/ENVOY has been reported in *T. reesei* as a PAS/LOV domain protein and has been proposed as a factor linking light responses with carbon source signaling and regulating light-dependent cellulase gene transcription [64,65]. Transcriptome analysis in *T. reesei* has also shown that BLR-1, BLR-2, and ENV1 are involved in the regulation of genes associated with D-galactose/pentose catabolism and glycoside hydrolases [66]. The increased expression of ENV1 in the present study suggests that downstream transcriptional adjustment may have been activated after blue-light perception, and this may also be linked to the selective expression changes in CAZyme-related candidate genes identified above.

### 4.5. Limitations and Future Directions

Although this study provides insights into the morphological and transcript-level responses of *T. harzianum* to blue LED illumination, several limitations should be acknowledged. However, because this study was conducted under a single-wavelength condition, it has limitations in fully reflecting diverse light intensities and spectral conditions, including red light, green light, mixed light, and natural light. A recent study in *T. guizhouense* reported that short-term blue-light exposure activated HOG1–ATF1 signaling and identified 824 blue-light-regulated genes by transcriptome analysis after 30 min of blue-light treatment [22]. Therefore, the analysis performed at a single time point on day 5 in the present study may not have captured transient expression changes occurring during the early response to blue LED exposure. Accordingly, the transcript abundance of profiles observed in this study may primarily reflect later-stage or cumulative responses following sustained blue LED exposure. Future time-course analyses involving RNA sampling at multiple time points after blue-light exposure are needed to distinguish early and late transcriptional responses. Another limitation is that the RNA-seq analysis was based on one pooled library per condition, which limited the statistical identification of differentially expressed genes. Future studies using independently sequenced biological replicates are needed to perform statistically supported differential expression analysis. In the case of white light, spectral composition can vary depending on the light source; therefore, future studies should evaluate the reproducibility of blue-light responses under white-light conditions with precisely defined spectra and correlated color temperatures, as well as under mixed-wavelength conditions.

In addition, although this study identified changes in the expression of specific gene groups under blue LED illumination through transcriptome analysis, functional validation was not performed to determine whether these genes directly contribute to the inhibition of mycelial growth and the promotion of sporulation. Future studies should clarify the causal relationships between blue LED-associated morphological changes and candidate genes by performing knockout, knockdown, overexpression, and complementation analyses targeting CAZyme candidate genes and photoreceptor that showed condition-associated expression differences under blue LED illumination.

## 5. Conclusions

This study investigated the growth, sporulation, and transcriptome expression patterns of *T. harzianum* under blue LED illumination. Under blue LED illumination, *T. harzianum* exhibited morphological changes characterized by inhibited mycelial growth and promoted sporulation compared with darkness. Transcriptome analysis also revealed condition-associated expression differences under blue LED illumination, indicating that the morphological responses were accompanied by changes in transcript abundance. Among the candidate genes, CAZyme-related candidates showed higher or lower expression under blue LED illumination, suggesting that light conditions may be linked to transcriptome expression differences associated with cell wall remodeling, carbohydrate metabolism, and sporulation. In addition, the increased expression of the photoreceptor-related genes BLR-1, BLR-2, and ENV1 supports the possibility that the morphological changes and CAZyme-related expression differences were associated with blue-light perception and regulatory responses.

By integratively presenting the morphological responses and transcriptomic expression patterns of *T. harzianum* under blue LED illumination, this study provides a phenotypic and molecular basis for future studies aimed at elucidating blue-light response mechanisms and characterizing the functions of candidate genes. From an industrial perspective, these findings suggest that light spectra in controlled cultivation environments may be associated with the growth and differentiation patterns of *Trichoderma*, and they provide foundational information for discussing facility-environment management and strategies to reduce contamination risk.

## Figures and Tables

**Table 1:** Primer information.

Gene	Primer	Sequence (5′–3′)
rpb2	fRPB2-5F	Forward: GAY GAY MGW GAT CAY TTY GG
	fRPB2-7cR	Reverse: CCC ATR GCT TGY TTR CCC AT

**Table 2:** Primers used in this study.

Name	Sequence (5′–3′)
GT 8	F: GAGATCGCTGAGACATGGGA
	R: GACAGCAGCTTACTTGGACG
GH 18	F: AGCACCTCCAATGGACATGA
	R: TGTGGGAAGATGGCATGGAT
AA 1	F: ATTCTGCCGCCTCTTCTTCT
	R: CACTTGCTGGTAGTCTGGGA
CE 9	F: GCATCATCTACTCGGTGGGA
	R: TGGGTTTCGGTGATGCAAAG
GH 16	F: ATGTTGTTCCAGCCCTCGTA
	R: GTCTCATTGTCGCCCGATTC
GT 35	F: TGCGGAAAACCTTGTCCTTG
	R: ACCCTCGTCTGTCTGAGTTG
GH 76	F: CACGATGAGACCTGCAATGG
	R: CCCACTCCCCATAACCAGTT
AA 3	F: GCATCACTGGCTTATCTGGC
	R: TTCGCCCTACAGAGCAAGAA
GT 20	F: CATTGCAAGAAAAGCTGCCG
	R: GCGTTGTGGAGGGAAATGTT
GT 2	F: TGAAACATCCGGGCAAACAA
	R: CTCCTGTGCTCTTCCGATCT
GT 31	F: TACCTGAAGCGTGATGGGTT
	R: TTGGGATCAAGGGCAGACAT
CE 5	F: AACTCGTTGATAGTGGGCCA
	R: CCTTTCCCTTTTCACAGCCC
BLR-1	F: TTCCTGCAAACGTGTTCTCG
	R: CCAGTCGTCGCATCTTTCAG
BLR-2	F: CCAGGACCAATTCAACACCG
	R: CGAGAATCTGCAGCAAGTCC
ENV1	F: CCCTGGTTTCTACGAGCTGA
	R: ACGGGCTCATGAATGTCTGA
Actin	F: TATCATGATCGGTATGGGTCAGAAGGACTCC
	R: CATCACAATGTTGCCGTAGAGCTCCTTTCGG

GT 8: glycosyltransferase family 8 protein; GH 18: glycoside hydrolase family 18 protein; AA 1: auxiliary activities family 1; CE 9: carbohydrate esterase family 9 protein; GH 16: glycoside hydrolase family 16 protein; GT 35: glycosyltransferase family 35 protein; GH 76: glycoside hydro-lase family 76 protein; AA 3: auxiliary activities family 3; GT 20: glycosyltransferase family 20 protein; GT 2: glycosyltransferase family 2 protein; GT 31: glycosyltransferase family 31 protein; CE 5: carbohydrate esterase family 5 protein.; BLR-1: blue-light regulator 1; BLR-2: blue-light regulator 2; ENV1: gene encoding the PAS/LOV-domain protein ENVOY.

**Table 3:** Representative enriched GO term among blue LED illumination responsive candidate genes in *T. harzianum*.

**Expression Pattern**	**Ontology**	**GO ID**	**GO Term**	**Count**	**Enrichment Ratio**	**FDR**
Higher in Blue	BP	GO:0051179	localization	581	1.278024	<0.001
Higher in Blue	BP	GO:0006810	transport	458	1.27114	<0.001
Higher in Blue	BP	GO:0070887	cellular response to chemical stimulus	228	1.345278	0.000436
Higher in Blue	BP	GO:0042908	xenobiotic transport	13	3.927067	0.002893
Higher in Blue	MF	GO:0008559	ABC-type xenobiotic transporter activity	13	3.927067	0.002893
Higher in Blue	MF	GO:0015405	ATPase-coupled transmembrane transporter activity	40	1.909209	0.006987
Lower in Blue	BP	GO:1900436	positive regulation of filamentous growth in response to starvation	11	13.13813	<0.001
Lower in Blue	BP	GO:0034413	ascospore wall beta-glucan biosynthetic process	5	83.60625	<0.001
Down in Blue	BP	GO:1990344	secondary cell septum biogenesis	5	83.60625	<0.001
Lower in Blue	BP	GO:0001676	long-chain fatty acid metabolic process	7	24.64184	<0.001
Lower in Blue	BP	GO:0070592	cell wall polysaccharide biosynthetic process	9	8.438762	0.000122
Lower in Blue	MF	GO:0003843	1,3-beta-D-glucan synthase activity	5	83.60625	<0.001
Lower in Blue	MF	GO:0035251	UDP-glucosyltransferase activity	6	13.0862	0.00045

Candidate genes were selected using |log2FC| ≥ 1 and max TPM ≥ 15. Higher in Blue and Lower in Blue indicate candidates with higher and lower TPM values under blue LED illumination, respectively. BP, biological process; MF, molecular function.

**Table 4:** Candidates genes with pronounced expression changes under blue LED illumination.

Gene ID	CBS226.95 Protein ID	Putative Function	Regulation	log_2_FC
TRINITY_DN1389_c0_g1	XP_024770744.1	glycosyltransferase family 8 protein	UP in blue	4.584
TRINITY_DN10486_c0_g1	XP_024778061.1	glycoside hydrolase family 18 protein	UP in blue	4.248
TRINITY_DN11803_c0_g1	XP_024776463.1	auxiliary activity family 1 multicopper oxidase	UP in blue	3.703
TRINITY_DN8553_c0_g1	XP_024775748.1	carbohydrate esterase family 9 protein	UP in blue	3.759
TRINITY_DN2376_c0_g1	XP_024777332.1	glycoside hydrolase family 16 protein	UP in blue	3.399
TRINITY_DN378_c0_g1	XP_024780719.1	glycosyltransferase family 35 protein	UP in blue	3.182
TRINITY_DN7689_c1_g1	XP_024769507.1	glycoside hydrolase family 76 protein	Down in blue	−5.967
TRINITY_DN443_c3_g1	XP_024768366.1	auxiliary activity family 3 GMC oxidoreductase	Down in blue	−7.324
TRINITY_DN6670_c0_g1	XP_024779890.1	glycosyltransferase family 20 protein	Down in blue	−5.314
TRINITY_DN3328_c0_g1	XP_024780942.1	glycosyltransferase family 2 protein	Down in blue	−3.462
TRINITY_DN609_c1_g1	XP_024773302.1	glycosyltransferase family 31 protein	Down in blue	−3.443
TRINITY_DN9694_c0_g1	XP_024777423.1	carbohydrate esterase family 5 protein	Down in blue	−4.860

Candidate genes were selected from the fold-change-based transcriptome analysis using a |log2FC| ≥ 3 threshold. Positive and negative log2FC values indicate increased and decreased expression under blue LED, respectively.

**Table 5:** Identification and transcript abundance profiles of photoreceptor-related genes under blue LED illumination.

Photoreceptor	Trinity Gene ID	Trinity Protein ID	CBS226.95 ID	Identity	TPM (Darkness/Blue)	Fold Change	Log2FC
BLR-1	TRIITY_DN4923_c0_g1	TRIITY_DN4923_c0_g1_i5.p1	M431DRAFT_83749	89.5	8.68/26.11	2.79	1.48
BLR-2	TRIITY_DN2550_c0_g1	TRIITY_DN2550_c0_g1_i25.p1	M431DRAFT_84653	90.9	11.69/23.06	1.89	0.92
ENV1	TRIITY_DN5078_c0_g1	TRIITY_DN5078_c0_g1_i14.p1	M431DRAFT_35611	71.7	126.82/538.34	4.20	2.07

## Data Availability

The RNA-seq data generated in this study have been deposited in the NCBI Gene Expression Omnibus (GEO) under accession number GSE334040. Other raw data are available from the authors upon reasonable request.

## Abbreviations

The following abbreviations are used in this manuscript:

GT 2	Glycosyltransferase family 2 protein
GT 8	Glycosyltransferase family 8 protein
GT20	Glycosyltransferase family 20 protein
GT31	Glycosyltransferase family 31 protein
GT32	Glycosyltransferase family 32 protein
GH16	Glycoside hydrolase family 16 protein
GH18	Glycoside hydrolase family 18 protein
GH76	Glycoside hydrolase family 76 protein
AA1	Auxiliary activities family 1
AA3	Auxiliary activities family 3
CE5	Carbohydrate esterase family 5 protein
CE9	Carbohydrate esterase family 9 protein
